# Sexuality in women recovered from COVID-19

**DOI:** 10.1192/j.eurpsy.2022.781

**Published:** 2022-09-01

**Authors:** M. Lagha, G. Hamdi, N. Dhaouadi, S. Chebli, R. Ridha

**Affiliations:** 1 Razi Hospital, Forensic Psychiatry, La Manouba, Tunisia; 2 Abderrahmen Mami Hospital, Preventive Medicine, Ariana, Tunisia

**Keywords:** sexuality, recovered, Covid-19, women

## Abstract

**Introduction:**

While several studies have assessed the impact of the COVID-19 pandemic on sexuality and sexual behavior in the general population, very few studies have assessed sexuality after Sars-Cov 2 infection.

**Objectives:**

The objective of our study were to assess sexuality in women recovered from COVID-19 and to compare it to healthy controls.

**Methods:**

This is a cross-sectional case-control study.

We randomly recruited women, from April 1st to 30th, 2021.The women in the case group have been infected with Sars-Cov 2, with a benign or pauci-symptomatic clinical form, and cured for one to two months at the time of the study without any post-COVID complications. Women included in the control group have not been infected with Sars-Cov 2. Sexuality was assessed by the Female Sexual Function Index (FSFI).

**Results:**

In total, we recruited 30 women in the case group and 30 women in the control group.The average age of the case group was 35.8 ±6.8 years versus an average age of 35.3 ± 6.33 years in the control group. The majority of coronavirus infections were symptomatic (83.3% (n=25)). An FSFI score <26.55 and corresponding to impaired sexual function was found in 63.33% of women in the case group (n=19), versus 53.33% of women in the control group (n=16) with a significant difference between the two groups (p=0.009)

**Conclusions:**

COVID-19 is significantly associated with sexual dysfunction, even in mild or pauci-symptomatic clinical forms.

**Disclosure:**

No significant relationships.

